# The EZH2 inhibitor GSK343 suppresses cancer stem-like phenotypes and reverses mesenchymal transition in glioma cells

**DOI:** 10.18632/oncotarget.21311

**Published:** 2017-09-27

**Authors:** Tianfu Yu, Yingyi Wang, Qi Hu, WeiNing Wu, Youzhi Wu, Wenjin Wei, Dongfeng Han, Yongping You, Ning Lin, Ning Liu

**Affiliations:** ^1^ Department of Neurosurgery, The First Affiliated Hospital of Nanjing Medical University, Nanjing, China; ^2^ Department of Neurosurgery, Nanjing First Hospital, Nanjing Medical University, Nanjing, China; ^3^ Department of Neurosurgery, The Affiliated Ganzhou Hospital of Nanchang University, Ganzhou, Jiangxi Province, China; ^4^ Department of Neurosurgery, Xuzhou Central Hospital, Xuzhou, China; ^5^ Department of Neurosurgery, The First People's Hospital Chuzhou, Chuzhou, China

**Keywords:** EZH2, EZH2 inhibitor, epigenetics, glioma, glioma stem cell

## Abstract

Enhancer of zeste homolog 2 (EZH2) is the catalytic unit of polycomb repressive complex 2 (PRC2) which epigenetically silences many genes involved in tumor-suppressive mechanisms via the trimethylation of lysine 27 of histone H3 (H3K27me3). We recently found that overexpression of EZH2 was associated with poor outcome of glioblastoma (GBM). In this study, we examined the antitumor effects of the EZH2 inhibitor GSK343 on glioma cells *in vitro* and *in vivo*. The proliferation and cell cycle of glioma cells was measured. Wound healing assay and transwell invasion assay were performed to evaluate the capacity of migration and invasion of glioma cells. Western blot, qPCR, immunoprecipitation and fluorescent staining were used to test the levels of EZH2 and associated proteins. Spheroid formation assay and clonogenic assays were conducted to assess the stemness of glioma stem cells. Finally, the effect of GSK343 was measured through a nude mice model with intracranially xenotransplanted glioma. We found that GSK343 reduced proliferation, attenuated cell motility and reversed epithelial-mesenchymal transition in U87 and LN229 glioma cells. GSK343 also suppressed the stemness of cell lines and patient derived glioma stem cells. Further, GSK343 inhibited histone H3K27 methylation and upregulated the expression of EZH2 target genes thereby regulating the levels of markers involved in epithelial-mesenchymal transition and stemness. Taken together, our results indicate that GSK343 could be a potential drug against glioblastoma.

## INTRODUCTION

The polycomb repressive complex 2 (PRC2), an epigenetic repressor, is a multi-subunit complex consisting of three core subunits including enhancer of zeste homolog 2 (EZH2), suppressor of zeste 12 (SUZ12), and embryonic ectoderm development (EED). EZH2, the catalytic component of PRC2, transfers a methyl group from S-adenosyl methionine (SAM) to lysine 27 of histone H3 (H3K27) through its SET domain. Cooperation with SUZ12 and EED is necessary for full performance of PRC2 histone lysine methyltransferase activity [1, 2]. Trimethylated H3K27 (H3K27me3) serves as a docking site for DNA methyltransferases and the monoubiquitylation of H2AK119 catalyzed by PRC1 [3, 4]. PRC2 silences many genes involved in cell proliferation, cell-cycle regulation, cell differentiation, and self-renewal [5–11]. Overexpression of EZH2 confers pro-oncogenic functions that are implicated in a broad spectrum of solid tumors [12–16].

We recently found that elevated EZH2 expression is associated with glioma grade and poorer overall survival [17]. Moreover, the depletion of EZH2 expression by RNA interference produced anti-glioma effects both *in vitro* and *in vivo* [18]. The relapse of malignant glioma after surgical resection is mainly because of the stem cell-like properties of a small number of cells, called glioma stem cells (GSCs). Furthermore, mesenchymal transition is associated with increased migratory capacity and invasiveness. GSC and mesenchymal transition are both found to facilitate the progression of glioma [19–24]. What's more, the functions of EZH2 are closely related with stemness and mesenchymal transition [25]. Thus, the development of drugs that can block EZH2 oncogenic activity may be promising. In this research, we mainly focused on the influence of GSK343 on glioma stemness and mesenchymal transition.

Three types of potent EZH2 inhibitors have been discovered, including 3-deazaneplanocin A (DZNep), SAM-competitive inhibitors (GSK343, GSK126, and EPZ-6438), and the stabilized α-helix of EZH2 peptide (SAH-EZH2) [26–28]. In this report, we examined the effects of GSK343, a promising SAM-competitive inhibitor, in glioma cells *in vitro* and *in vivo*.

## RESULTS

### GSK343 suppresses cell proliferation, migration and invasion and induces G_0_/G_1_ arrest in glioma cells

**Figure 1:**
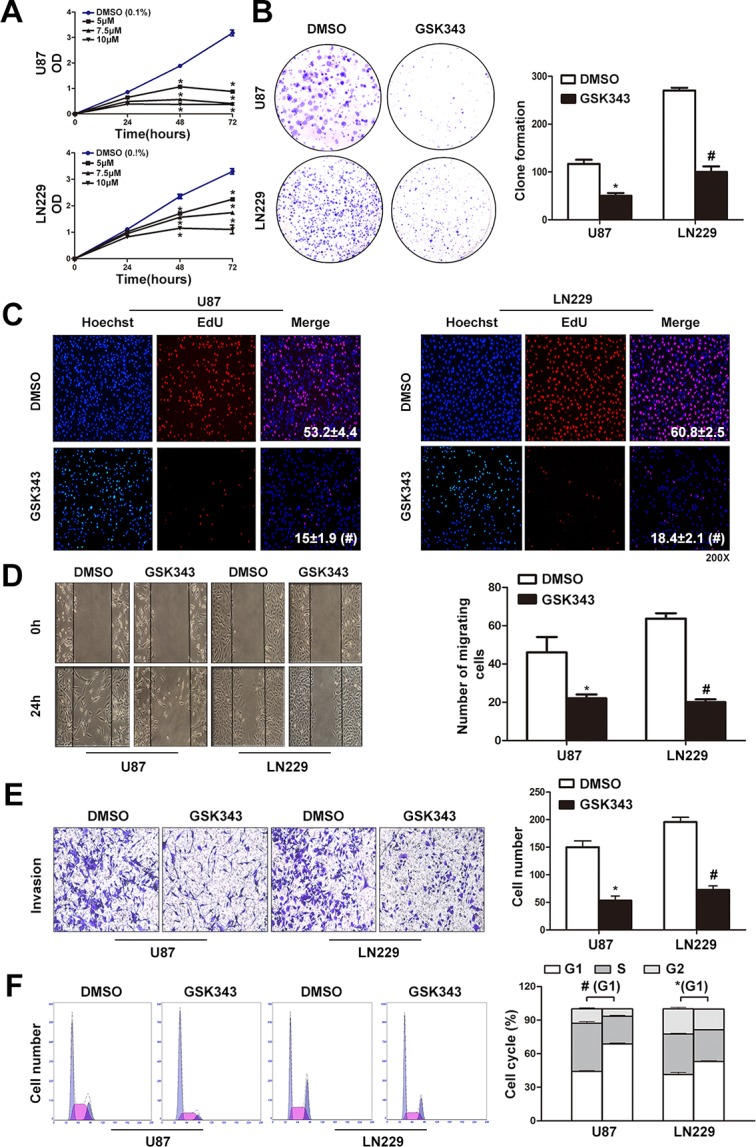
GSK343 suppresses cell proliferation, migration and invasion and induces G_0_/G_1_ arrest in glioma cells **(A)** Dose-dependent effects of GSK343 (5 μM, 7.5 μM, and 10 μM) on cell proliferation over time in U87 and LN229 cells. Cell growth was measured by the cell counting kit-8 (^*^p < 0.05, ^#^*p* < 0.001). **(B)** Colony formation ability of U87 and LN229 cells was decreased following 5 μM GSK343 treatment (^*^p < 0.05, ^#^*p* < 0.001). **(C)** Red fluorescence means cells in the S phase and blue fluorescence represents all of the cell nuclei. Representative profiles of EdU (red) and Hoechst 33342 (blue) staining in the culture treated with 5 μM GSK343 or 0.1% DMSO 48 h were shown. Rate of DNA synthesis of GSK343-treated cells was lower. **(D, E)** Cell migration and invasion ability of U87 and LN229 cells which were measured by wound-healing and transwell invasion assays was reduced after 5 μM GSK343 treatment for 24 h (^*^p < 0.05, ^#^*p* < 0.001). **(F)** Treatment of 5 μM GSK343 in U87 and LN229 cells for 48 h induced cell-cycle arrest in G_0_/G_1_ phase (^*^p < 0.05, ^#^*p* < 0.001).

We first examined the cell viability of glioma cells after treated with different concentrations of GSK343 (5 μM, 7.5 μM and 10 μM) or vehicle control (0.1% DMSO) for 24 h, 48 h and 72 h. As shown in Figure 1A, GSK343 significantly inhibited cell proliferation of both U87 and LN229 glioma cells in time- and dose-dependent manners. CCK8 assay revealed that 5 μM was the half maximal inhibitory concentration (IC50) of GSK343 in glioma cells (Supplementary Figure 3E). Colony formation was also reduced after GSK343 treatment (Figure 1B). The DNA synthesis of glioma cells under the influence of GSK343 was assessed via EdU assay. The EdU proliferation assay was performed after treated with 5μM GSK343 or 0.1% DMSO for 48 h. The percentage of EdU-positive glioma cells treated with GSK343 was significantly lower than cells treated with DMSO (Figure 1C). We next performed wound-healing and transwell invasion assays. A tendency toward decreased capacity of migration and invasion was seen in both cell lines treated with GSK343 (Figure 1D and 1E). In addition, flow cytometry analysis revealed that GSK343 treatment led to the accumulation of cells in G_0_/G_1_ phase and a reduction in S phase cells (Figure 1F). Finally, we tested the effect of GSK343 on TJ905 which is a low EZH2 expression cell line [17] (Supplementary Figure 1A). Although the protein levels of EZH2 and H3K27me3 were both down-regulated, GSK343 had little impact on the function of TJ905 glioma cells (Supplementary Figure 1B-1E). These results indicated that GSK343 was an EZH2-dependent inhibitor.

### GSK343 inhibits histone H3K27 methylation and up-regulates the expression of EZH2 target genes

**Figure 2:**
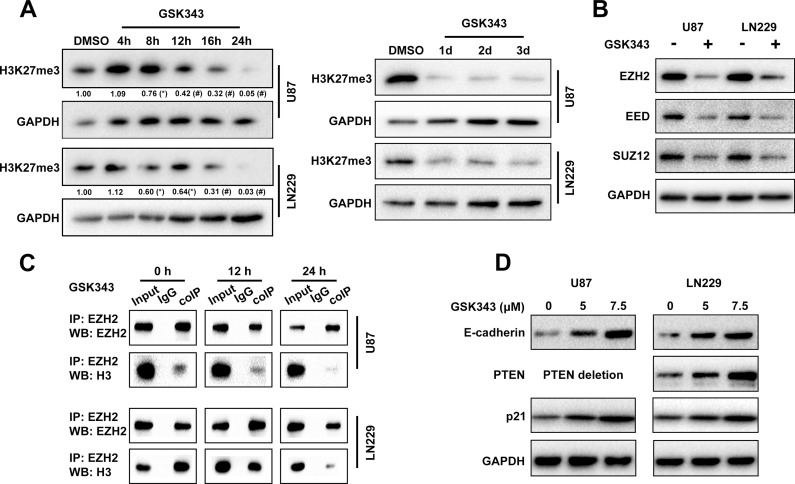
GSK343 treatment leads to downregulation of H3K27me3 and increases expression of E-cadherin, p21 and PTEN **(A)** Expression of H3K27me3 in U87 and LN229 cells treated with 5 μM GSK343 over time was determined by western blot analysis. GADPH was used as a loading control. **(B)** U87 and LN229 cells were treated with 5 μM GSK343 or 0.1% DMSO for 48 h and the levels of EZH2, EED, SUZ12, and GAPDH were examined by western blot analysis. **(C)** Co-immunoprecipitation analyses of EZH2-H3 complex formation in U87 and LN229 cells after treatment with 5 μM GSK343 for 12 h or 24 h. **(D)** Western blot analysis of E-cadherin, p21, and PTEN expression in cells treated with 5 μM, 7.5μM GSK343 and 0.1% DMSO for 48 h.

GSK343 functions as a SAM-competitive EZH2 inhibitor. We next performed a time-course analysis to investigate H3K27 methylation following 5 μM GSK343 treatments. Our data showed that the level of H3K27 methylation was down-regulated as early as 8 h after GSK343 treatment and decreased levels lasted for 3 days (Figure 2A). Because EZH2 mediated methylation of Histone 3 is based on the integrity of the PRC2 multi-subunit complex and the interaction between EZH2 and H3, the status of EZH2, EED, SUZ12 and H3 was examined. Notably, GSK343 treatment caused a reduction in EZH2, EED, and SUZ12 protein levels (Figure 2B). Furthermore, immunoprecipitation experiment demonstrated a significant time-dependent dissociation of the protein interaction between EZH2 and H3 (Figure 2C). Finally, to examine the mechanisms underlying the reversion of malignant characteristics by GSK343, we evaluated the expression levels of some classical EZH2 target genes including E-cadherin, PTEN, and p21 by western blot analysis. After treatment with different concentration of GSK343 (0, 5 and 7.5 μM) for 48 h, the protein expression levels of these tumor suppressor genes were elevated (Figure 2D). We found that the efficiency of GSK343 is dose-dependent. The U87 cells have homozygous deletion of *PTEN* thus PTEN level could not be compared.

### GSK343 reverses mesenchymal transition in glioma cells

**Figure 3:**
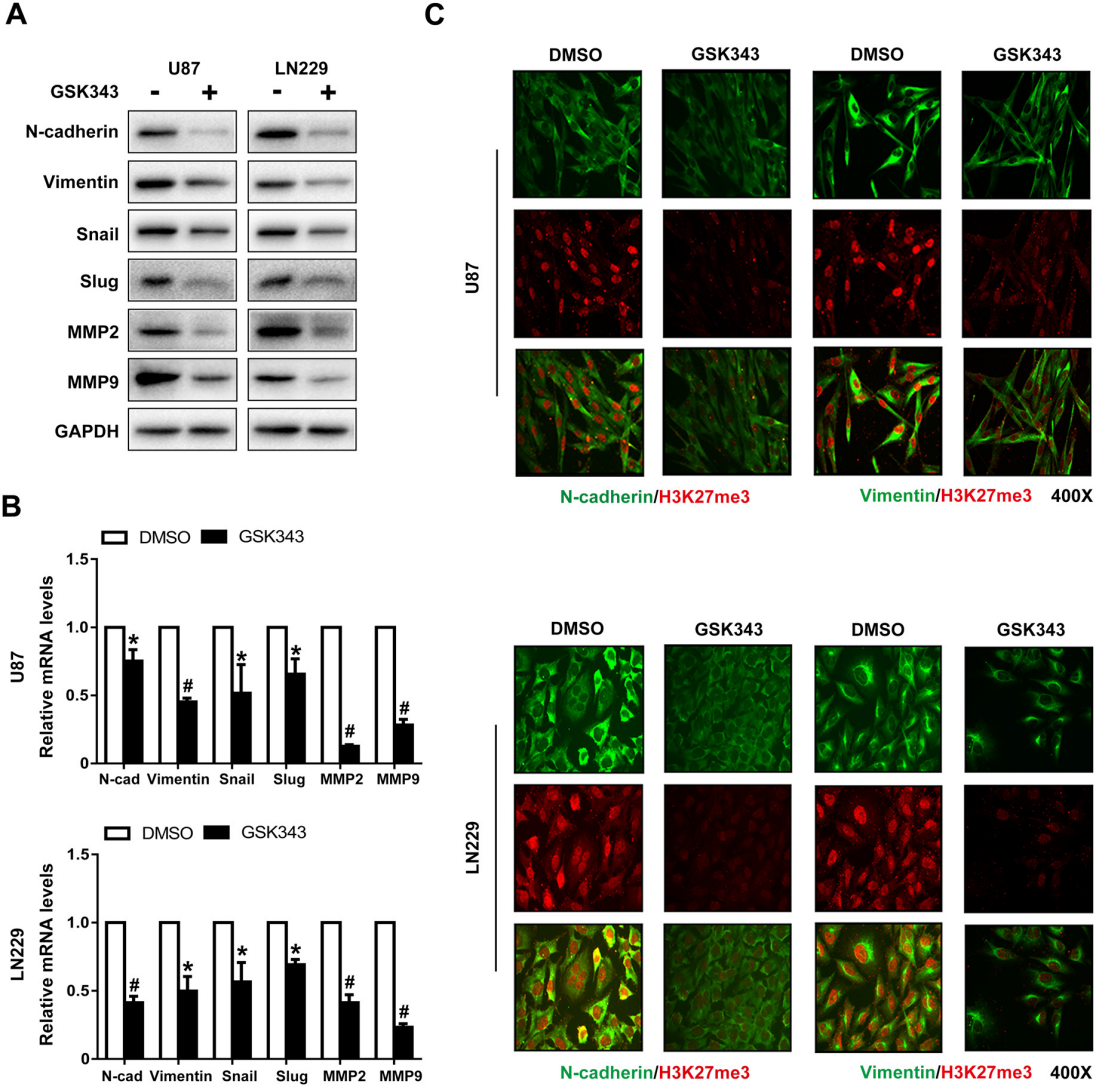
GSK343 suppresses mesenchymal transition in U87 and LN229 cells **(A, B)** Protein and mRNA levels of N-cadherin, Vimentin, MMP2, MMP9, Snail, Slug, and GAPDH in U87 and LN229 cells treated with 5 μM GSK343 or 0.1% DMSO for 48 h were examined by western blot analysis and RT-PCR (^*^p < 0.05, ^#^*p* < 0.001). **(C)** Immunofluorescence staining of N-cadherin (green), Vimentin (green), and H3K27me3 (red) in U87 and LN229 cells after treatment with 5 μM GSK343 or 0.1% DMSO for 48 h.

The levels of mesenchymal markers and transcription factors including N-cadherin, Vimentin, MMP2, MMP9, Snail and Slug were examined in U87 and LN229 cells. The expression of these proteins was downregulated following 5 μM GSK343 treatments (Figure 3A and 3B). We also performed immunofluorescence analysis of H3K27me3, N-cadherin, and Vimentin in glioma cells. We observed that the decrease of N-cadherin and Vimentin was accompanied by the decline of H3K27me3 in cells treated with GSK343 (Figure 3C).

### GSK343 treatment attenuates the stemness of glioma stem cells

**Figure 4:**
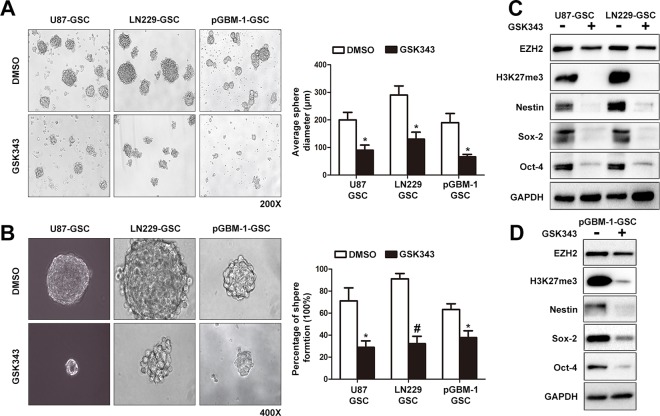
GSK343 suppressed cancer stem cell associated phenotypes of glioma cells **(A)** Spheroid formation assay was used for assessing the stemness of GSCs treated with 5 μM GSK343 or 0.1% DMSO for 5 d. Representative photomicrographs of sphere formation assay (left) and statistical analysis of average diameter of spheres (right) (^*^p < 0.05). **(B)** Clonogenic assay was used to measure the stemness of GSCs treated with 5 μM GSK343 or 0.1% DMSO for 15 d. Representative photomicrographs of new clonal sphere (left) and statistical analysis of the percentage of positive wells (right) (^*^p < 0.05). **(C)** Protein levels of Nestin, Sox-2, Oct-4, EZH2, and H3K27me3 in GCSs treated with 5 μM GSK343 or 0.1% DMSO for 5 d. **(D)** The expression of Nestin, Sox-2, Oct-4, EZH2 and H3K27me3 in pGBM-1 derived GCSs treated with 5 μM GSK343 or 0.1% DMSO for 5 d was tested by western blot assay.

Considering the limitations of traditional cell lines in stemness experiments, we derived primary cells (pGBM-1) from an adult primary GBM sample. GSK343 inhibited the proliferation of pGBM-1 cells and reduced the protein levels of EZH2 and H3K27me3 in pGBM-1 cells (Supplementary Figure 2). Then we examined the impact of GSK343 on GSCs derived from U87, LN229 and pGBM-1, including sphere-forming capacity and the expression of GSC markers. Treatment of spheres with 5 μM GSK343 for 5 days caused a reduction in the diameter and a decrease in the number of tumor spheres (Figure 4A). We further tested the effect of GSK343 on stemness through a clonogenic assay. Disaggregated spheres were seeded in 96-well plates at clonal density (1 cell per well) and cultured in sphere medium added with 5 μM GSK343 or 0.1% DMSO. After 15 days, the percentage of positive wells (positive well means 1 cell colony/well at least) in GSK343 treated GSCs was lower than DMSO treated GSCs and the spheres in GSK343 treated wells were smaller than DMSO treated wells (Figure 4B). Then, we examined levels of the pluripotency factors including Nestin, Sox-2, and Oct-4 to determine the influence of GSK343 on GSCs. We observed a significant decline of Nestin, Sox-2, Oct-4, and H3K27me3 in GSCs treated with GSK343 (Figure 4C and 4D). However, unlike findings from adherent GBM cells, there was no change in the level of EZH2 in GSCs after GSK343 treatment (Supplementary Figure 3A).

### GSK343 inhibits tumor growth *in vivo*

**Figure 5:**
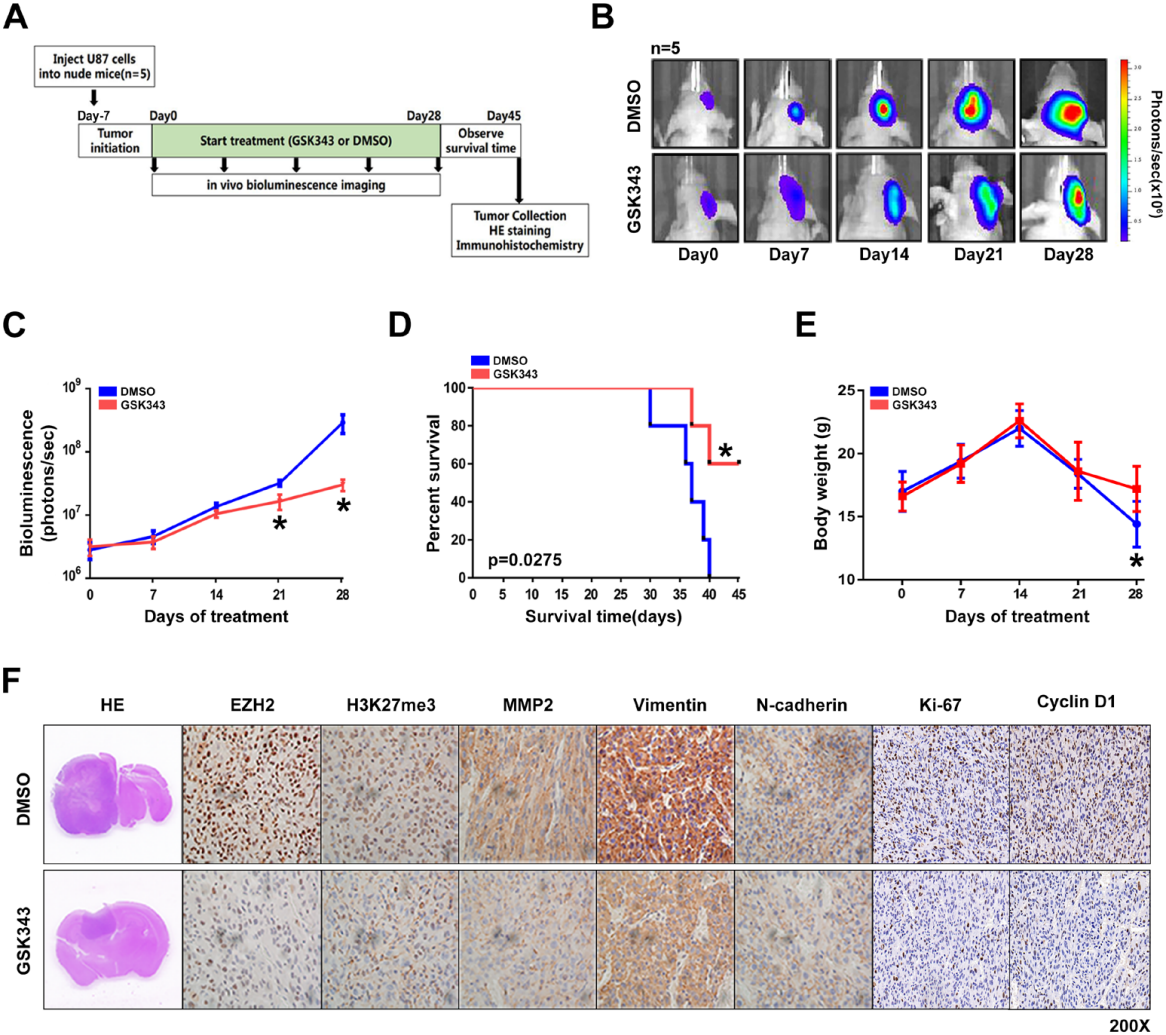
GSK343 inhibits glioma tumorigenesis and invasiveness *in vivo* **(A)** The panel shows a schematic presentation of *in vivo* experiment. **(B, C)** After the beginning of treatment, nude mice (n=5) were monitored by luciferase live imaging system weekly. The total amount of output photons of each mouse was collected to evaluate the tumor sizes of the GSK343-treatment group and the DMSO-treatment group (^*^p < 0.05). **(D)** Kaplan–Meier analysis of overall survival of the GSK343-treatment group and the DMSO-treatment group (^*^p < 0.05). **(E)** Nude Weight of the GSK343-treatment group and the DMSO-treatment group (^*^p < 0.05).s **(F)** Immunohistochemistry on xenografted tumors from both the GSK343-treatment group and the DMSO-treatment group.

**Figure 6:**
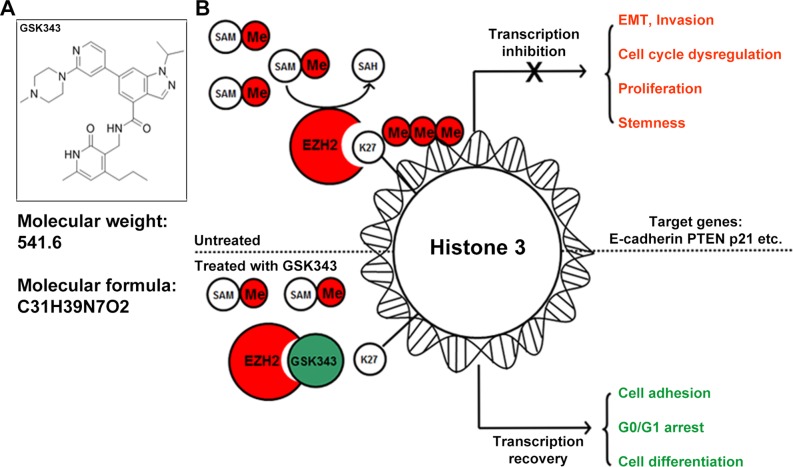
The basic information and functions of GSK343 **(A)** The chemical construction and molecular weight of GSK343. **(B)** The panel shows a schematic presentation of the mechanism of GSK343.

We next examined the effect of GSK343 on glioma cells *in vivo*. Nude mice were intracranially xenotransplanted with luciferase-expressing U87 glioma cells. After tumor formation, nude mice were treated with intraperitoneal injections of GSK343 (10 mg/kg in 200 μL PBS) or diluted DMSO (10 μL in 200 μL PBS) every other day (Figure 5A). After the start of treatment (7 days), tumor growth was monitored by a live animal bioluminescence imaging system every week (Figure 5B). Results showed that tumors treated with GSK343 exhibited significantly slower growth from day 21 and onwards (Figure 5B and 5C). After 28 days of treatment with GSK343 or DMSO every other day, we continued to observe the survival of mice. At the end of observation (day 45), the survival was analyzed by Kaplan–Meier curves. Compared with the control group, the GKS343-treated group showed prolonged survival period (Figure 5D). The nude weight was shown in Figure 5E. Consist with the results obtained from the *in vitro* study, immune-histochemical analyses demonstrated that the expression levels of EZH2, H3K27me3, MMP2, Vimentin, N-cadherin, ki-67 and Cyclin D1 were downregulated in tumor specimens from the GSK343-treated group (Figure 5F and Supplementary Figure 3F). The basic information and functions of GSK343 were summarized in Figure 6.

## DISCUSSION

Epigenetic abnormalities have been proven to play a pivotal role in tumor progression. These alterations are distinct from common genetic alterations such as mutations or amplification because epigenetic regulation can be reversed by enzyme inhibitors including histone deacetylases, histone methyltransferases, and DNA methyltransferases [29]. The reversible nature of epigenetic changes provides an opportunity for the development of specific inhibitors that will be a benefit to targeted cancer therapies.

Three different strategies have been used for the design of EZH2 inhibitors. The most recent strategy involves targeted disruption of the PRC2 by SAH-EZH peptides that selectively disrupt the EZH2/EED complex. The traditional method blocks the removal of S-adenosyl-L-homocysteine (SAH), the metabolite of methylation. SAH hydrolase is responsible for the clearance of SAH and sustains the methyltransferase activity. The SAH hydrolase inhibitor DZNep causes the accumulation of SAH, which in turn leads to by-product inhibition of the SAM-dependent methyltransferase activity such as EZH2 [26]. However, mounting evidence indicates that DZNep lacks specificity [30]. Because SAM is the universal methyl donor for histone methyltransferases reaction, SAM-competitive inhibitors have been the main approach for EZH2 inhibitors owing to their high selectivity to EZH2. This kind of inhibitors include GSK126, a potent drug against lymphoma, and EPZ-6438 which is the first orally bioavailable inhibitor and is currently in phase I/II clinical trials [28, 31, 32]. GSK343, another SAM-competitive EZH2 inhibitor, is over 1000-fold selective for other histone methyltransferases and 60-fold selective over EZH1. It effectively inhibits the proliferation of several solid tumors such as epithelial ovarian cancer and cervical cancer. GSK343 also acts as an autophagy inducer in breast cancer cells and enhanced the sensitivity of sorafenib in hepatocellular carcinoma cells [33–36]. Our results showed that GSK343 suppressed cell proliferation and invasion in glioma cells. Most previous studies were only focused on the basic functions of these SAM-competitive inhibitors, and therefore further studies to decipher underlying mechanisms, particularly in glioma treatment, are needed.

Apart from H3K27 methylation, EZH2 also functions as an oncogene activator through an uncanonical pathway: the methylation of non-histone proteins. In glioma cells, EZH2 binds to and methylates STAT3, leading to enhanced STAT3 activity by increased tyrosine phosphorylation of STAT3, especially in GSC [37]. We found that the treatment of GSK343 in glioma cells significantly decreased H3K27 methylation and co-precipitation with EZH2-H3 in a time-dependent manner and reduced the levels of core units of PRC2. Our previous data showed that EZH2 knockdown triggered a reduction of STAT3 expression, whereas inhibition of STAT3 activity by WP1066 decreased EZH2 expression [17]. Besides STAT3, c-Myc enforces the overexpression of EZH2 through transcriptional and post-transcriptional mechanisms as well [38–40]. Interestingly, the expression of c-Myc is also positively regulated by EZH2 in GBM [18]. We assumed that there exists an EZH2/c-myc positive feedback (Supplementary Figure 3D). We found that in normal glioma cells, these two oncogenic transcription factors, c-myc and EZH2, increased the protein level of each other. Furthermore, GSK343 treatment in normal glioma cells not only decreased the protein level of EZH2, but also depressed the expression of c-myc. In GSCs, however, things have changed. GSK343 only reduced the protein level of EZH2. This also occurred when si-EZH2 was used (Supplementary Figure 3B). Furthermore, after the co-incubation of EZH2-plasimd and si-cmyc, the expression of c-myc did not change in GSCs (Supplementary Figure 3C). We think that the possible mechanism is that the upstream positive regulatory factors of c-myc in GSCs are more complex both in species and numbers (Supplementary Figure 3D). These data suggest that EZH2 may contribute to glioma tumor progression by H3K27 trimethylation-dependent and STAT3-/c-Myc- dependent pathways.

Glioma stem-like cells are responsible for the initiation, propagation, and recurrence of glioma [41]. Recently, epithelial-to-mesenchymal transition (EMT) was found to facilitate the progression of GSCs. Snail enhances irradiation-induced self-renewal of glioma cells via EMT. In addition, Twist1, an EMT inducer, was recently shown to co-regulate glioma stemness with Sox2 [42, 43]. Since glial and glioma cells are not of an epithelial origin, the term epithelial-to-mesenchymal transition is inappropriate. We prefer to call it mesenchymal transition or EMT-like transition in title and text [44]. Based on the link between EMT-like transition and glioma stemness, we assumed that GSK343 not only reversed EMT-like transition but also attenuated glioma stem cell-like phenotypes. In our study, GSK343 significantly impaired the capacity of sphere formation in GSC and also abrogated the levels of H3K27me3 and GSC markers including Nestin, Sox-2, and Oct-4. Taken together, these data indicate that GSK343 might be a promising GSC-specific treatment.

Our present study demonstrates that GSK343, an inhibitor of EZH2, suppresses the proliferation, invasion, and cancer stem-like phenotypes and reverses mesenchymal transition of glioma cells *in vitro* and *in vivo*. These findings suggest that GSK343 could be an important tool to understand the oncogenic role of EZH2 in glioma and may be a viable approach for the treatment of glioma.

## MATERIALS AND METHODS

### EZH2 inhibitor and cell culture

The EZH2 inhibitor GSK343 was purchased from Sigma-Aldrich and was used at a final concentration of 5-10 μM. The human glioblastoma cell lines U87 and LN229 were purchased from Chinese Academy and the TJ905 GBM cell line was established and characterized by the laboratory of Neuro-oncology of the Tianjin Neurological Institute. Cell lines were routinely tested for absence Mycoplasma. Primary GBM cells (pGBM-1) that derived from a GBM surgical specimen were maintained in DMEM supplemented with 10% FBS.

### Glioma stem cell culture, spheroid formation assay and clonogenic assays

To culture glioma stem cells (GSC), single-cell populations which came from adherent U87, LN229 and pGBM-1 cells, were resuspended in DMEM/F12 (Gibco), supplemented with N2(1/100, Invitrogen), B27(1/50, Invitrogen), 10 ng/ml recombinant human basic fibroblast growth factor (FGF, Invitrogen) and 10 ng/ml recombinant human epidermal growth factor (EGF, Invitrogen). This GSC culture medium was called sphere medium for short. After the primary spheroids achieving a diameter >200 μm, to further enrich CSC, spheres were dissociated with TrypLETM Express (Gibco) and resuspended in sphere medium. Following five rounds of sphere culture, these secondary cellular spheroids were considered as enriched in GSC and were used for next experiments. For spheroid formation assay, disaggregated spheres were placed in six-well plates and were cultured in sphere medium added with 5 μM GSK343 or 0.1% DMSO. A week later, we counted the diameter of spheres which bigger than 25 μm. For clonogenic assays, disaggregated spheres were seeded in 96-well plates at clonal density (1 cell per well) and cultured in sphere medium added with 5 μM GSK343 or 0.1% DMSO. To renew growth factors supply, fresh sphere medium was added every 5 d. 15 d later, we counted the percentage of spheres formation.

### Cell counting kit-8 assay

U87, LN229, TJ905 and pGBM-1 cells (2,000 cells/well) were plated in 96-well plates in triplicate and were incubated at 37°C overnight. Subsequently, the cells were treated with GSK343 (5 μM, 7.5 μM, 10 μM) or vehicle control (0.1% DMSO) for 24 h, 48 h and 72 h. Finally, the cell growth was measured by CCK-8 Cell Counting Kit (Dojindo, Japan) following the manufacturer's protocols.

### Colony formation assay

U87 and LN229 cells were seeded at a density of 500 cells per well in separate 60-mm tissue culture plates. After treated with 5 μM GSK343 or vehicle control for 2 weeks, colonies were fixed with 4% methanol for 30min and subsequently stained with 0.1% crystal violet for 30 min. The number of colonies was counted to determine the colony-forming efficiency.

### 5-ethynyl-29-deoxyuridine (EdU) proliferation assay

To assess the proliferative activity of U87 and LN229 glioma cells, Click-iT EdU Alexa Fluor 594 Imaging Kit (Thermo Fisher Scientific, Massachusetts, USA; Cat. No. C10339) was used according to the manufacturer's protocol. After treated with 5 μM GSK343 or 0.1% DMSO for 48h, the proportion of glioma cells incorporated EdU was determined with fluorescence microscopy (Nikon, Tokyo, Japan) at x200 magnification.

### Wound healing assay

U87 and LN229 cells were seeded in 6-well plates and were cultured until they reached 100% confluence. Next, a ten-microliter sterile pipette tip was used to create scratches across the cell monolayer and the photographs of scratched areas were taken. Then, cells were treated with 5 μM GSK343 or vehicle control. 24 h later, a total of nine scratched areas were selected randomly in each well and were photographed under inverted microscope (Nikon, Tokyo, Japan). The cells protruding from the border of the scratches were counted.

### Transwell invasion assay

24-well plates using transwell inserts (Corning, New York, USA) which were pre-coated with 20 μg/μL Matrigel (BD Biosciences, New Jersey, USA) were used to measure cell invasion. The upper chambers were added with 50,000 U87, LN229 and TJ905 cells which were treated with 5 μM GSK343 or 0.1% DMSO and were dissolved in 200 μL serum-free media. In parallel, 900 μL DMEM media with 10% FBS was added to lower chamber of each well. After incubation for 24 h at 37°C, cells from the upper surface of the membrane were removed with a cotton swab and the penetrated cells were fixed with 4% methanol for 5 min and then stained with 0.1% crystal violet for 30 min. Six fields of cells were captured and counted randomly under inverted microscope (Nikon, Tokyo, Japan) at x200 magnification in each well.

### Flow cytometry analysis

After treated with GSK343 for 48 h, U87, LN229 and TJ905 cells were harvested and washed with phosphate-buffered saline (PBS). Then, 75% ethanol was used to fix cells at −20°C overnight. Next, DNA in cells was stained by Hank's balanced salt solution including 50 mg/mL propidium iodide and 50 mg/mL RNaseA for 1 h at room temperature. Finally, cell cycle of these cells was analyzed by Gallios flow cytometer (Beckman Countler).

### RNA extraction and quantitative real-time PCR

RNA was extracted from U87 and LN229 by TRIzol reagent (Invitrogen, Darmstadt, Germany). TaqMan real-time reverse transcription PCR was carried out for synthesizing cDNA by ABI StepOnePlus system (Applied Biosystems, Massachusetts, USA). Then, quantitative real-time PCR analysis was performed using ABI PRISM 7500 FAST Real-TIME PCR System. The relative expression was determined through the CT method and the results were normalized to GAPDH expression. There followed the primer sequences used in RT-PCR.

GAPDH, forward 5’-GCACCGTCAAGGCTGA GAAC-3’ and reverse 5’-TGGTGAAGACGCCAG TGGA-3’; N-cadherin, forward 5’-TATGCCCAAGACAA AGAGACC-3’ and reverse 5’-CAACTTCTGCTGACT CCTTCA-3’; Vimentin, forward 5’-TGAGTACCGGA GAC AGGTGCAG-3’ and reverse 5’-TAGCAGCTTCAACGGCAAAGTTC-3’; MMP2, forward 5’-GCTATGGACTTGGGAGAA-3’ and reverse 5’-TGGAACGGAATGGAAAC-3’; Snail, forward 5’-TTTACCTTCCAGCAGCCCTA-3’ and reverse 5’-GACAGAGTCCCAGATGAGCA-3’; Slug, forward 5’-TTCGGACCCACACATTACCT-3’ and reverse 5’-GCAGTGAGGGCAAGAAAAAG-3’.

### Western blot analysis

RIPA lysis buffer (KeyGEN, Jiangsu, China) was used to extract proteins from cells. Equal amounts of protein were separated by SDS-PAGE, followed by electrotransfer onto polyvinylidene difluoride membranes (Thermo Fisher Scientific, Massachusetts, USA). Then, membranes were blocked for 2 h with 5% nonfat milk and then incubated at room temperature with primary antibody. After incubating with secondary antibody for 2 h, membranes were developed using an enhanced chemiluminescence detection system (GE Healthcare, Chicago, USA). Immunoblot analysis used the following primary antibodies: EZH2, SUZ12, PTEN, p21, E-cadherin, N-cadherin, Vimentin, MMP2, Snail, Slug and GAPDH were obtained from Cell Signaling Technology (Massachusetts, USA). H3K27me3, H3, EED were purchased from Abcam (Cambridge, UK).

### Immunoprecipitation

After treated with GSK343, cells were collected and lysed using lysis buffer supplemented with PMSF. Then, equal amounts of protein was subjected was subjected to anti-EZH2 antibody (CST, Massachusetts, USA) following overnight incubation at 4°C. Following this, protein-antibody immunoprecipitates were collected by protein A/G plus-agarose (Santa Cruz Biotechnology, Texas, USA). Finally, the EZH2 and H3 protein was analyzed by Western blotting.

### Fluorescent staining

Cells fixed by 4% formalin were incubated with mouse monoclonal anti-H3K27me3 (Abcam, Cambridge, UK), rabbit monoclonal anti-N-cadherin or rabbit monoclonal anti-Vimentin (CST, Massachusetts, USA) overnight at 4°C and then with Alexa 488- or 568-labeled anti-mouse or anti-rabbit IgG antibody (Thermo Fisher Scientific, Massachusetts, USA) 2 h at room temperature. After treated with DAPI (Beyotime Biotechnology, Jiangsu, China) for 10 min, cells were examined with Zeiss axiophot photomicroscope (Carl Zeiss AG, Jena, Germany).

### *In vivo* experiments, IHC, and H&E staining

Animal experiments were approved by the Animal Management Rule of the Chinese Ministry of Health (documentation 55, 2001) and were in accordance with the approved guidelines and the experimental protocol of Nanjing Medical University. 6-week-old female nude mice purchased from Cancer Institute of the Chinese Academy of Medical Science were orthotopically implanted with 1×10^6^ luciferase-expressing U87 cells per mouse (GSK343-treated group, N=5; DMSO-treated group, N=5). After 7 days, nude mice received either vehicle (10 μL DMSO in 200 μL PBS) or GSK343 (10 mg/kg in 200 μL PBS) intraperitoneally every other days. Tumor growth was assessed weekly by live animal bioluminescence imaging system. Mice were euthanized when moribund. Whole brains were removed. Then brains were fixed in 4% formalin and were embedded in paraffin. Next, tissue sections were incubated with antibodies including EZH2, H3K27me3, N-cadherin, Vimentin and MMP2 or stained with H&E. The percentage of positive cells of each section was analyzed at x200 magnification.

### Statistical analysis

To analyze differences in each two-group comparison, t-test was employed while one-way ANOVA was performed to determine the difference among at least three groups. Kaplan–Meier analysis was used to assess the survival rate of mice. P<0.05 was considered statistically significant.

## SUPPLEMENTARY MATERIALS FIGURES



## Abbreviations

OD: optical density
PRC2: polycomb repressive complex 2
EZH2: zeste homolog 2
SUZ12: suppressor of zeste 12
EED: embryonic ectoderm development
SAM: S-adenosyl methionine
H3K27: lysine 27 of histone H3
DZNep: 3-deazaneplanocin A
EMT: epithelial-mesenchymal transition
GSC: glioma stem cell.
