# Supplementary material for: The EZH2 inhibitor GSK343 suppresses cancer stem-like phenotypes and reverses mesenchymal transition in glioma cells
Source: Oncotarget. 2017 Sep 27;8(58):98348–59. doi: 10.18632/oncotarget.21311 (PMC5716734; doi:10.18632/oncotarget.21311)
Supplement: Supplementary file 1 [file oncotarget-08-98348-s001.pdf]

The EZH2 inhibitor GSK343 suppresses cancer stem-like phenotypes and reverses mesenchymal transition in glioma cells

SUPPLEMENTARY MATERIALS

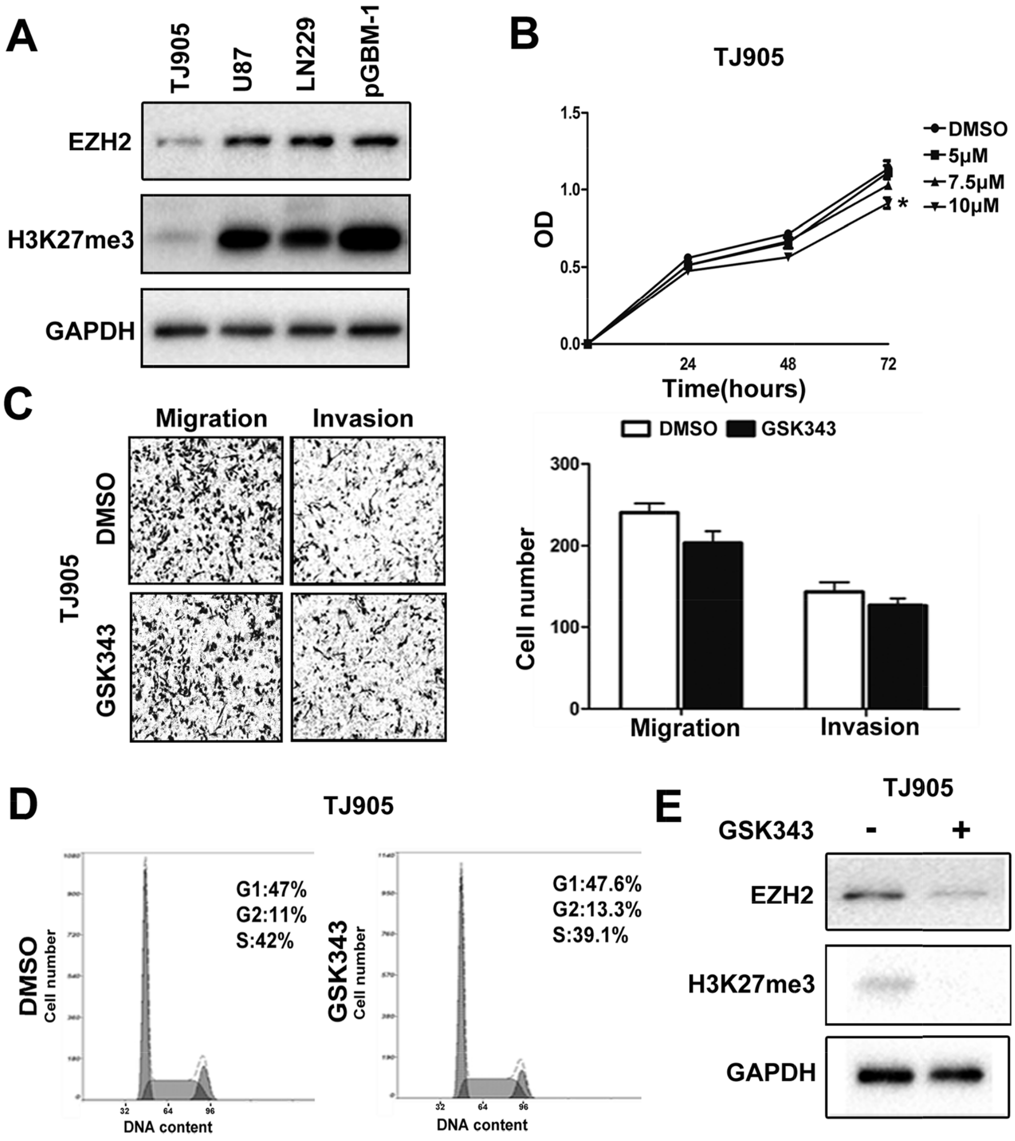

Supplementary Figure 1: The effects of GSK343 on cell growth, migration, invasion and cell cycle in TJ905 cells. (A) The protein levels of EZH2 and H3K27me3 in different GBM cell lines (TJ905, U87, LN229 and pGBM-1). **(B)** Dose-dependent effect of GSK343 (5 µM, 7.5 µM, and 10 µM) on cell proliferation over time in TJ905 cells. Cell growth was measured by the cell counting kit-8. TJ905 cells treated with 10 µM GSK343 for 72 h showed a reduced proliferation rate compared to cells treated with 0.1% DMSO ($^{*}p < 0.05$). **(C)** The treatment of TJ905 cells with 5 µM GSK343 for 24 h did not affect the migration and invasion ability of these cells. **(D)** The treatment of TJ905 cells with 5 µM GSK343 for 24 h did not influence cell cycle. **(E)** TJ905 cells were treated with 5 µM GSK343 or 0.1% DMSO for 48 h and the levels of EZH2 and H3K27me3 were examined by western blot analysis.

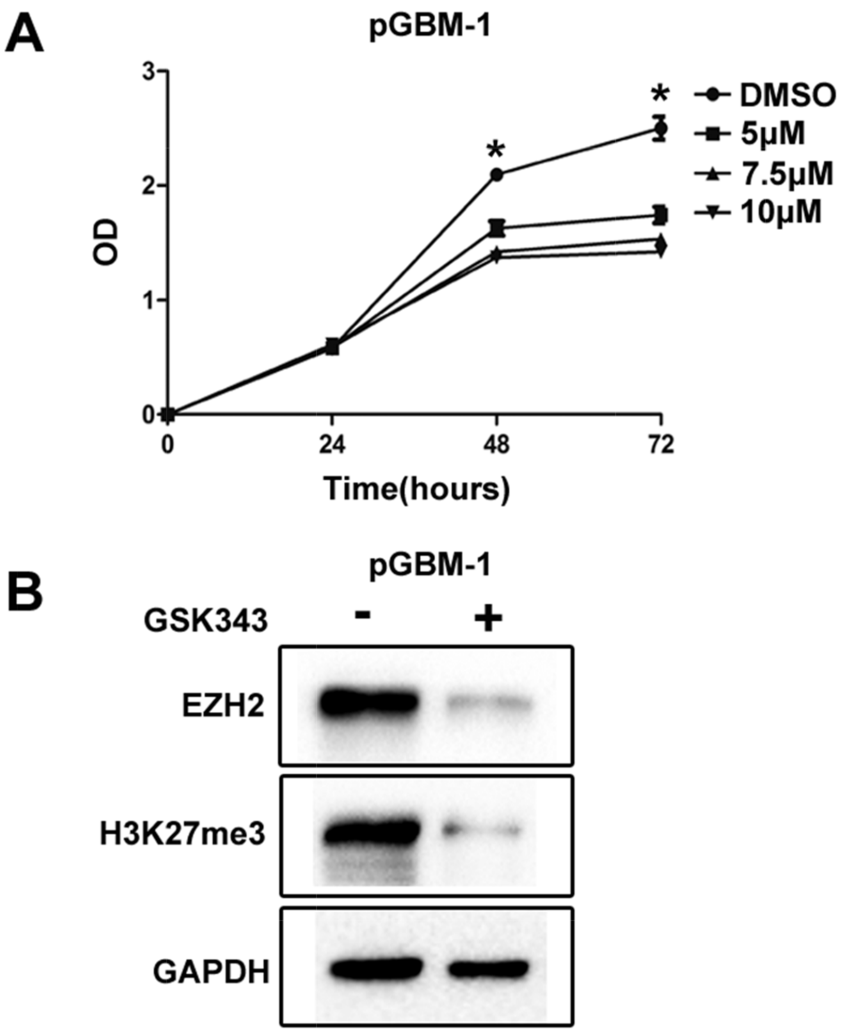

Supplementary Figure 2: GSK343 reduced proliferation rate and down-regulated protein levels of EZH2 and H3K27me3 in pGBM-1 cells. **(A)** Dose-dependent effect of GSK343 (5 μM, 7.5 μM, and 10 μM) on cell proliferation over time in pGBM-1 cells ($^*p < 0.05$). **(B)** pGBM-1 cells were treated with 5 μM GSK343 or 0.1% DMSO for 48 h and the levels of EZH2 and H3K27me3 were examined by western blot analysis.

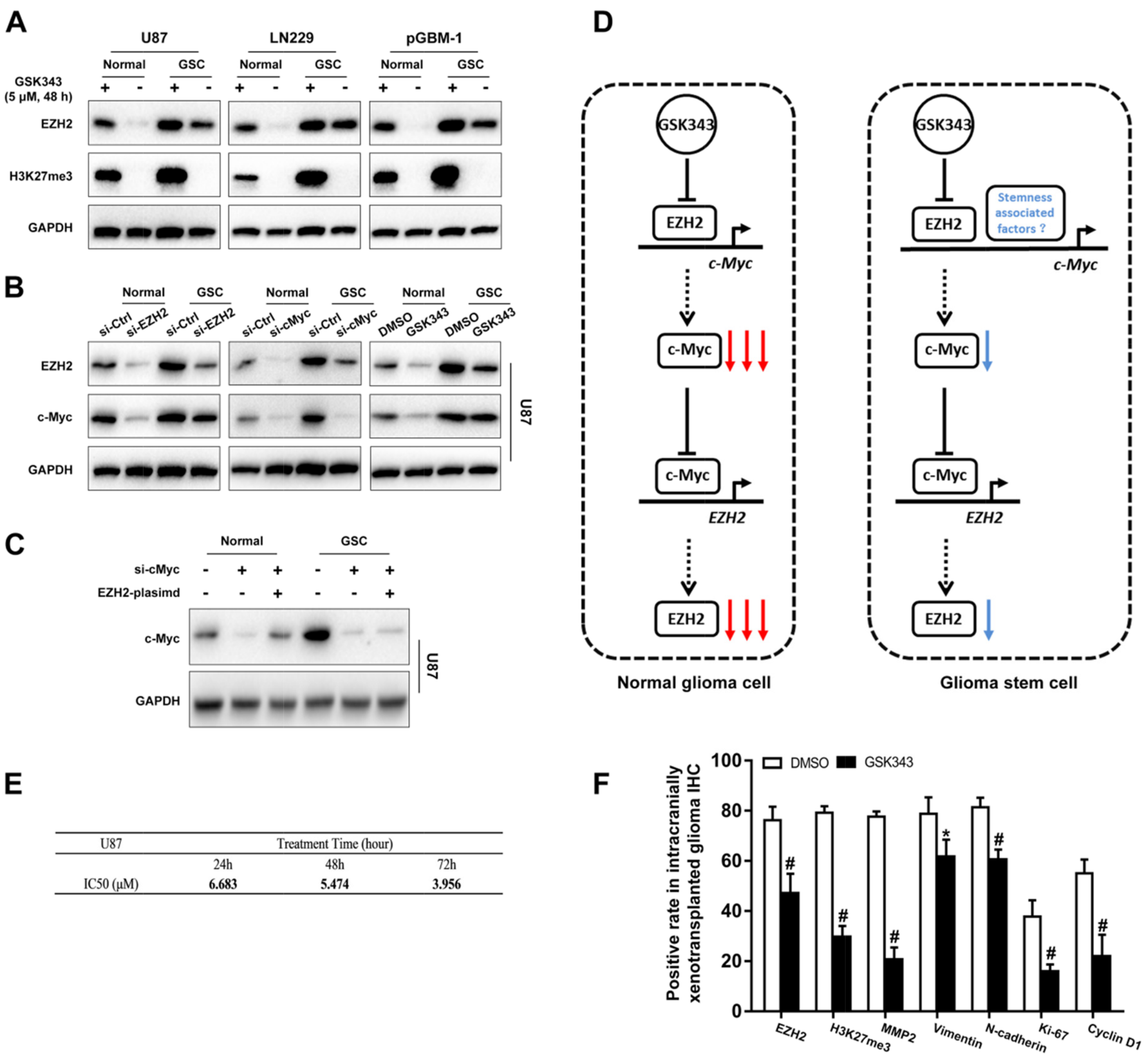

Supplementary Figure 3: A further study about the phenomenon which GSK343 did not decrease the protein level of EZH2 in GSCs. **(A)** The protein levels of EZH2 and H3K27me3 in GSK343 treated cells were shown. **(B)** The efficiency of si-EZH2, si-cMyc and GSK343 on EZH2 and c-Myc protein levels was illustrated. **(C)** After co-incubation with si-cMyc and EZH2-plasimd, c-Myc expression in U87 normal or stem cells was shown. **(D)** The schematic abstract may be able to explain that GSK343 did not decrease the protein level. **(E)** The half maximal inhibitory concentration (IC50) of GSK343 in U87 glioma cells was shown. **(F)** The positive rate of indicated protein shown in Fig. 5F was displayed. ($^{*}p < 0.05$, $^{\#}p < 0.001$).
